# Hedonic hunger and eating-disorder symptoms in relation to glycaemic control across treatment regimens in adults with type 2 diabetes: a cross-sectional study

**DOI:** 10.3389/fpsyg.2026.1872938

**Published:** 2026-10-28

**Authors:** Hakan Toğuç, Arjin Ayçiçek, Deniz Tamar, Büşra Hökelek, Gürkan Özden

**Affiliations:** 1 Department of Nutrition and Dietetics, Faculty of Health Sciences, Inonu University, Malatya, Türkiye; 2 Department of Nutrition and Dietetics, Mehmet Akif Inan Education and Research Hospital, Şanlıurfa, Türkiye; 3 Department of Nursing, Faculty of Nursing, Inonu University, Malatya, Türkiye

**Keywords:** eating behaviours, glycaemic control, hedonic hunger, medical treatment, type 2 diabetes

## Abstract

**Background:**

Eating-related behavioural factors may contribute to the complexity of glycaemic management in type 2 diabetes mellitus (T2DM), but their relationships with treatment context remain insufficiently understood. This study examined hedonic hunger and eating-disorder symptom dimensions in relation to glycaemic control across different treatment regimens in adults with T2DM.

**Methods:**

This cross-sectional study included 203 adults with T2DM attending an outpatient diet clinic in southeastern Turkey between March and September 2025. Participants were receiving oral antidiabetic medication only (*n* = 108), insulin only (*n* = 32), or combined oral medication plus insulin therapy (*n* = 63). Hedonic hunger was assessed using the Power of Food Scale (PFS), and eating-disorder symptom dimensions using the 13-item Eating Disorder Examination Questionnaire–Short Form (EDE-Q-13). Anthropometric measurements, 24-h dietary recall data, fasting plasma glucose (FPG), and glycated haemoglobin (HbA1c) were obtained. Hierarchical linear regression was used to examine factors associated with HbA1c.

**Results:**

HbA1c differed substantially across treatment groups and was highest in the insulin-only group (*p* < 0.001). Several PFS subscales and EDE-Q-13 symptom dimensions also differed between groups, although their effect sizes were small. Selected PFS dimensions were weakly positively correlated with glycaemic indicators, whereas the EDE-Q-13 eating-restraint subscale and total score showed weak inverse correlations with HbA1c. In the fully adjusted model, insulin-only and combination therapy were associated with higher HbA1c compared with oral medication only, while body mass index (BMI) was inversely associated with HbA1c. Neither the PFS food available subscale nor the EDE-Q-13 total score remained independently associated with HbA1c. The final model explained 27.2% of the variance in HbA1c (adjusted *r*^2^ = 0.230).

**Conclusion:**

Treatment context was more strongly related to glycaemic status than the behavioural measures assessed, although modest differences in hedonic hunger and eating-disorder symptom dimensions were evident across treatment regimens. These findings suggest that eating-related behavioural characteristics may provide complementary information in nutritional assessment and should be interpreted alongside the broader clinical context of T2DM management.

## 1 Introduction

Type 2 diabetes mellitus (T2DM) is a chronic metabolic disorder characterized by insulin resistance, progressive impairment of insulin secretion, and disturbances in glucose metabolism. Its global prevalence continues to increase, with approximately 589 million adults currently living with diabetes (International Diabetes Federation, 2025). Inadequate glycaemic control contributes to cardiovascular disease, nephropathy, retinopathy, neuropathy, and other diabetes-related complications. Fasting plasma glucose (FPG) and glycated haemoglobin (HbA1c) are therefore widely used indicators of glycaemic status and treatment effectiveness (American Diabetes Association, 2021). The American Diabetes Association recommends an HbA1c target of <7.0% for many non-pregnant adults while emphasizing individualization according to clinical characteristics, treatment burden, and hypoglycaemia risk (American Diabetes Association Professional Practice Committee, 2024).

Although pharmacological treatment is central to T2DM management, achieving and maintaining glycaemic targets also depends on sustained dietary self-regulation. Eating behaviour is influenced not only by nutritional knowledge and physiological hunger but also by psychological processes, reward sensitivity, emotional regulation, cognitive restraint, body-related concerns, and environmental exposure to palatable foods. Traditionally, appetite regulation has been viewed primarily through a homeostatic framework involving energy balance, hunger, and satiety. However, eating may also be driven by hedonic mechanisms operating partly independently of immediate physiological energy requirements (Vijayashankar et al., 2024).

Hedonic hunger refers to the desire or motivation to consume highly palatable foods in the absence of an immediate caloric deficit (Lowe et al., 2009; Lowe and Butryn, 2007). It reflects the psychological influence of the food environment and reward-related responsiveness to food cues. Highly palatable foods, particularly those rich in fat and/or sugar, may activate reward pathways and increase motivation to eat even when energy needs have been met (Lowe and Butryn, 2007; Espel-Huynh et al., 2018). Hedonic hunger is commonly assessed using the Power of Food Scale (PFS), which evaluates responsiveness to palatable food when it is available, physically present, or recently tasted (Lowe et al., 2009). The Turkish version of the PFS has demonstrated satisfactory validity and reliability (Ulker et al., 2021).

Emerging evidence supports a potential relationship between hedonic hunger and metabolic outcomes in T2DM. Cheung et al. (2018) reported an association between hedonic hunger and glycaemic control, particularly among individuals with obesity. In addition, reductions in hedonic hunger during a structured weight-management intervention were associated with improvements in weight-related and glycaemic outcomes (Schulte et al., 2020). Although these findings do not establish causality, they suggest that responsiveness to food-related reward cues may represent an additional behavioural dimension relevant to diabetes management. Eating-related behaviour in T2DM also encompasses restraint, body dissatisfaction, shape and weight concerns, bingeing, and other eating-disorder symptom dimensions. External, emotional, and uncontrolled eating patterns have been associated with poorer glycaemic control and dietary quality (Gal et al., 2024; Freitag et al., 2025). Dietary restraint is particularly complex in diabetes because it may reflect either adaptive dietary monitoring or rigid and potentially maladaptive restriction.

Treatment regimen may provide an additional clinical context in which these behavioural characteristics differ. Metformin may influence appetite-regulatory pathways and has been associated with increases in the appetite-suppressing metabolite N-lactoyl-phenylalanine (Foretz et al., 2019; Scott et al., 2024). Insulin therapy may affect eating behaviour through mechanisms related to hypoglycaemia prevention and treatment, potentially altering food-related decision making and carbohydrate consumption (Przezak et al., 2022). Nevertheless, treatment-related changes in appetite should not be equated directly with hedonic hunger. Moreover, treatment regimen in observational populations is not randomly assigned and may reflect disease duration, previous glycaemic control, disease progression, treatment burden, and clinical indication. Thus, behavioural differences between treatment groups may reflect broader clinical characteristics rather than direct pharmacological effects.

Despite increasing interest in behavioural determinants of diabetes outcomes, important gaps remain. Previous studies have mainly examined hedonic hunger in relation to obesity, weight-management interventions, or overall glycaemic control (Cheung et al., 2018; Schulte et al., 2020), while other studies have assessed individual eating behaviours without explicitly considering routine treatment regimens (Gal et al., 2024; Freitag et al., 2025). Comparatively little is known about whether hedonic hunger and eating-disorder symptom dimensions differ among adults receiving oral antidiabetic medication alone, insulin alone, or combined therapy. Evidence from non-Western clinical populations is also limited. Sociocultural differences in food exposure, dietary norms, body-related attitudes, and diabetes self-management may affect these relationships, supporting the need for evidence from Turkish clinical populations.

Accordingly, this study aimed to examine hedonic hunger and eating-disorder symptom dimensions in relation to glycaemic control across different treatment regimens in adults with T2DM. Specifically, we compared PFS and EDE-Q-13 scores among individuals receiving oral antidiabetic medication only, insulin only, or combined oral medication plus insulin therapy and examined their associations with FPG and HbA1c. We hypothesized that greater hedonic hunger, particularly responsiveness to food availability, would be associated with poorer glycaemic control. We also examined whether overall eating-disorder symptom severity was associated with HbA1c after adjustment for relevant demographic and clinical factors. Given the limited evidence supporting specific directional differences across treatment regimens, between-group comparisons of PFS and EDE-Q-13 dimensions were considered exploratory.

## 2 Materials and methods

### 2.1 Study design and setting

This cross-sectional study was conducted in the outpatient diet clinic of a state hospital in southeastern Turkey between March and September 2025. Recruitment was restricted to adults attending the outpatient clinic, and hospitalized patients were not eligible for participation. A non-probability, volunteer-based sampling approach was used. Potentially eligible outpatients were informed about the study, and those who agreed to participate and provided written informed consent were enrolled.

### 2.2 Participants and eligibility criteria

Adults aged 18 years or older who attended the outpatient diet clinic and had a documented diagnosis of type 2 diabetes mellitus were assessed for eligibility. The diagnosis of type 2 diabetes was verified using the hospital information system. Participants were eligible if they were receiving routine medical treatment for type 2 diabetes and were clinically stable at the time of the study assessment. Hospitalized patients were not eligible for inclusion. Four participants in the insulin-only group had been diagnosed within the preceding 15 days; all were clinically stable outpatients with no acute illness, diabetic ketoacidosis, or recent hospitalization.

Participants were excluded if they had chronic kidney disease, liver disease, or another serious chronic condition that could substantially affect nutritional status or glycaemic control. Individuals with a clinically diagnosed eating disorder, those currently using glucagon-like peptide-1 receptor agonists, and those using psychiatric or psychotropic medications were also excluded. Although individuals with a previously diagnosed eating disorder were excluded, subclinical eating-disorder symptom dimensions were evaluated using the EDE-Q-13.

A total of 203 eligible individuals agreed to participate, provided written informed consent, and were enrolled in the study. Complete data were available for all 203 participants, and all enrolled participants were included in the analyses. A formal screening log was not maintained for potentially eligible individuals who declined participation or for individuals excluded before enrolment; therefore, the total number approached and the reasons for non-participation could not be reported.

### 2.3 Sample size

The sample size was estimated *a priori* using a one-way ANOVA model based on between-group differences in HbA1c, which was considered a clinically meaningful glycaemic outcome for comparing treatment groups. Using data from Cheung et al. (2018) as the reference for HbA1c, the required minimum sample size was calculated as 147 participants, assuming an effect size of *f* = 0.26, an alpha level of 0.05, and 80% statistical power (Cheung et al., 2018). After allowing for an approximate 5% missing-data rate, the target sample size was set at 155 participants. Ultimately, 203 adults with T2DM were included in the study.

### 2.4 Treatment-group classification and study assessments

Participants were categorized into three mutually exclusive groups according to their current diabetes treatment regimen recorded in the hospital information system: oral antidiabetic medication only, insulin therapy only, and combined oral antidiabetic medication plus insulin therapy. Accordingly, 108 participants were receiving oral antidiabetic medication only, 32 were receiving insulin therapy only, and 63 were receiving combined oral antidiabetic medication plus insulin therapy.

A research dietitian administered the general information form, the Power of Food Scale (PFS), and the Eating Disorder Examination Questionnaire–Short Form (EDE-Q-13). Anthropometric measurements, including body weight and height, were obtained using standardized procedures. Glycaemic control indicators, including FPG and HbA1c, were retrieved from the participants’ medical records. Laboratory values recorded within 3 days of the questionnaire and anthropometric assessment were used in the analyses.

### 2.5 Data collection and variables

Data were collected during a single face-to-face outpatient assessment using a structured general information form, dietary assessment, anthropometric measurements, validated psychometric instruments, and laboratory records. The general information form included age, sex, educational level, perceived income status, smoking status, duration of diabetes, and current diabetes treatment regimen. Demographic and lifestyle information was obtained from the participants, whereas diabetes diagnosis, current treatment regimen, and laboratory findings were verified using the hospital information system. Treatment information was additionally confirmed by participant self-report when clarification was required.

Diabetes duration was recorded categorically rather than as an exact continuous duration. Because exact duration in months or years was not consistently available and the distribution was highly unbalanced, diabetes duration was operationally classified as ≤6 months and >6 months for the present analyses. This classification reflected the available data structure and should not be interpreted as a validated clinical threshold for diabetes chronicity.

Participants were classified into three mutually exclusive treatment groups, as described in Section 2.4: oral antidiabetic medication only, insulin therapy only, and combined oral antidiabetic medication plus insulin therapy. Information on specific oral antidiabetic drug classes and insulin types was not recorded; therefore, treatment exposure was analysed only at the broader regimen level.

### 2.6 Dietary assessment

Dietary intake was assessed using a single 24-h dietary recall administered face-to-face by a research dietitian. Participants were asked to report all foods, beverages, and other liquids consumed during the previous 24 h, including meal times, portion sizes, preparation methods, and ingredients where applicable. To improve the accuracy of portion-size estimation, standard measures and food portion photographs were used during the interview.

Energy and macronutrient intakes were calculated using BEBIS 8.1 nutritional analysis software, based on the food composition database integrated into the programme. Total daily energy intake, carbohydrate intake, protein intake, fat intake, and water consumption were included in the analyses. Because dietary intake was assessed using a single 24-h recall, the dietary data were interpreted as estimates of recent intake rather than habitual intake.

### 2.7 Anthropometric measurements

Anthropometric measurements were performed by the research dietitian using standardized procedures. Body weight was measured to the nearest 0.1 kg using a calibrated digital scale, with participants barefoot and wearing light clothing. Height was measured to the nearest 0.1 cm using a stadiometer, with participants standing barefoot, feet together, and head positioned in the Frankfurt plane.

Body mass index (BMI) was calculated as body weight in kilograms divided by height in metres squared (kg/m^2^). BMI was analysed as a continuous variable in group comparisons and regression analyses.

### 2.8 Power of food scale

Hedonic hunger was assessed using the Turkish version of the Power of Food Scale (PFS-Tr). The PFS was originally developed to assess appetite-related thoughts, feelings, and motivations elicited by highly palatable foods in food-abundant environments, independently of actual food consumption (Lowe et al., 2009). The original instrument comprises 15 items distributed across three food-proximity domains: food available, food present, and food tasted. The Turkish validation study retained 13 items after removing items 5 and 13 because of their high error covariance within the food available factor (Ulker et al., 2021).

Using the original 15-item numbering, the Turkish version comprises the food available subscale (items 1, 2, 10, and 11), the food present subscale (items 3, 4, 6, and 7), and the food tasted subscale (items 8, 9, 12, 14, and 15). The food available subscale reflects responsiveness to palatable foods that are generally accessible but not physically present; the food present subscale reflects responsiveness to foods that are physically present but have not yet been tasted; and the food tasted subscale reflects the pleasure and motivational response elicited when food is first tasted. Each item is rated on a 5-point Likert-type scale ranging from 1 (“do not agree at all”) to 5 (“strongly agree”). Subscale scores were calculated as the arithmetic mean of the items belonging to the corresponding subscale, and the total PFS-Tr score was calculated as the mean of all 13 items. Thus, subscale and total scores ranged from 1 to 5, with higher scores indicating greater hedonic responsiveness to palatable food cues. In the present study, Cronbach’s alpha for the total PFS-Tr was 0.935, indicating high internal consistency.

### 2.9 Eating disorder examination questionnaire–short form

Eating-disorder symptom dimensions were assessed using the Turkish version of the 13-item Eating Disorder Examination Questionnaire–Short Form (EDE-Q-13). The EDE-Q-13 was developed as a shorter version of the original EDE-Q that retains the principal cognitive and behavioural dimensions of eating-disorder psychopathology while incorporating bingeing and purging symptoms into the scored structure. Its Turkish version has demonstrated acceptable construct validity and internal consistency in adults (Esin and Ayyildiz, 2022).

The EDE-Q-13 assesses eating-disorder symptoms experienced during the preceding 28 days and comprises five subscales. Eating restraint consists of items 1–3; shape and weight over-evaluation consists of items 4 and 5; body dissatisfaction consists of items 6 and 7; bingeing consists of items 8–10; and purging consists of items 11–13. The bingeing and purging items are included in the scoring of the EDE-Q-13 rather than being recorded only as separate open-ended behavioural frequencies.

All items are scored on a 7-point scale ranging from 0 to 6. Depending on the item, the response categories represent symptom frequency or severity over the preceding 28 days, with higher values indicating more frequent or more severe symptoms. Subscale scores were calculated as the arithmetic mean of the items assigned to each subscale, and the total EDE-Q-13 score was calculated as the mean of all 13 items. Accordingly, both subscale and total scores ranged from 0 to 6, with higher scores indicating greater eating-disorder symptom severity.

The EDE-Q-13 was used as a dimensional measure of eating-disorder symptoms and not as a diagnostic instrument. Participants with a previously diagnosed eating disorder were excluded, but subclinical symptom dimensions were retained for analysis. In the present study, Cronbach’s alpha for the total EDE-Q-13 was 0.900, indicating high internal consistency (Esin and Ayyildiz, 2022).

### 2.10 Glycaemic control indicators

Glycaemic control indicators included FPG and glycated haemoglobin. FPG was reported in mg/dL, and HbA1c was reported as a percentage. Both parameters were obtained from the hospital electronic medical records.

In accordance with routine clinical procedures, venous blood samples for FPG were collected in the morning after an overnight fast of at least 8 h and analysed in the hospital central biochemistry laboratory using standardized automated methods. HbA1c values were obtained from EDTA-anticoagulated whole blood samples analysed as part of routine outpatient follow-up.

For the present study, laboratory values recorded within 3 days of the questionnaire and anthropometric assessment were used in the analyses. All biochemical analyses were performed in the hospital biochemistry laboratory in accordance with routine internal and external quality control procedures.

### 2.11 Statistical analysis

Dietary intake data were analysed using BEBIS 8.1 software, and statistical analyses were performed using IBM SPSS Statistics version 25.0. Continuous variables were assessed for distribution using the Kolmogorov–Smirnov test and Q–Q plots and were presented as median and interquartile range; categorical variables were presented as *n* (%). Between-group comparisons of continuous variables were performed using the Kruskal–Wallis H test, followed by Bonferroni-adjusted pairwise comparisons when significant. Categorical variables were compared using the chi-square test; Monte Carlo procedures were used when expected cell counts were insufficient. Significant categorical variables were further examined using two-sided Fisher’s exact tests with Bonferroni correction. Effect sizes were reported as epsilon-squared (*ε*^2^) and Cramer’s *V*, as appropriate. Associations between PFS and EDE-Q-13 scores and glycaemic control indicators were examined using Spearman’s rank correlation coefficients.

Hierarchical linear regression was performed with HbA1c as the dependent variable. Model 1 included treatment regimen, age, sex, BMI, and diabetes duration (≤6 months vs. > 6 months). Despite its unbalanced distribution, diabetes duration was retained in the multivariable model because of its potential clinical relevance to both treatment regimen and glycaemic control and, consequently, its potential role as a confounding factor. Model 2 additionally included smoking status and educational level; Model 3 added the PFS food available subscale; and Model 4 added the EDE-Q-13 total score. Age, sex, BMI, and diabetes duration were included as core demographic and clinical covariates, whereas smoking status and educational level were subsequently entered as potential confounders because they differed across treatment groups. The PFS food available subscale was entered as the hypothesis-driven hedonic-hunger variable, followed by the EDE-Q-13 total score as an overall measure of eating-disorder symptom severity. For regression analysis, educational level was recoded into three categories: illiterate, primary education, and secondary education or higher. Secondary education and university education or higher were combined because only six participants were in the university-or-higher category, and retaining this category separately would have resulted in a sparse subgroup and potentially unstable regression estimates. Oral medication only, female sex, non-smoking status, and illiteracy were used as reference categories. Regression assumptions were evaluated using residual plots, the Breusch–Pagan test, and variance inflation factors. Because heteroscedasticity was detected, HC3 robust standard errors and 95% confidence intervals were used. Model fit was evaluated using *r*^2^, adjusted *r*^2^, and changes in *r*^2^ (Δ*r*^2^). Statistical significance was set at *p* < 0.05.

## 3 Results

### 3.1 Demographic and clinical characteristics

A total of 203 adults with T2DM were included in the study. Of these, 108 participants were receiving oral antidiabetic medication only, 32 were receiving insulin therapy only, and 63 were receiving combined oral antidiabetic medication plus insulin therapy. Demographic and clinical characteristics according to treatment regimen are presented in Table 1.

**Table 1:** Demographic and clinical characteristics according to diabetes treatment regimen.

Variable	Category	Oral medication med (IQR)	Insulin only med (IQR)	Combination therapy med (IQR)	Total med (IQR)	*p*	Effect size
Age, years	Median (IQR)	62.00 (55.00–67.00)	56.50 (55.00–64.75)	60.00 (52.00–66.00)	60.00 (54.00–66.00)	0.400	0.000
Sex	Male	38 (35.2)	14 (43.8)	19 (30.2)	71 (35.0)	0.421	0.092
	Female	70 (64.8)	18 (56.2)	44 (69.8)	132 (65.0)		
Income status	Income less than expenditure	23 (21.3)	12 (37.5)	12 (19.0)	47 (23.2)	0.257	0.114
	Income equal to expenditure	60 (55.6)	16 (50.0)	35 (55.6)	111 (54.7)		
	Income more than expenditure	25 (23.1)	4 (12.5)	16 (25.4)	45 (22.2)		
Educational level	Illiterate	59 (54.6)	12 (37.5)	34 (54.0)	105 (51.7)	0.003	0.220
	Primary education	42 (38.9)	10 (31.2)	25 (39.7)	77 (37.9)		
	Secondary education	5 (4.6)^a^	8 (25.0)ᵇ	2 (3.2)ᵃ	15 (7.4)		
	University and above	2 (1.9)	2 (6.2)	2 (3.2)	6 (3.0)		
Smoking status	Yes	27 (25.0)ᵃ	14 (43.8)ᵃ	6 (9.5)ᵇ	47 (23.2)	<0.001	0.266
	No	81 (75.0)ᵃ	18 (56.2)ᵃ	57 (90.5)ᵇ	156 (76.8)		
Duration of diabetes	≤6 months	7 (6.5)	4 (12.5)	2 (3.2)	13 (6.4)	0.214	0.123
	>6 months	101 (93.5)	28 (87.5)	61 (96.8)	190 (93.6)		

Continuous variables are presented as median (interquartile range), and categorical variables as *n* (%). Different superscript letters within the same category row indicate statistically significant pairwise differences between treatment groups after Bonferroni adjustment; groups sharing the same letter do not differ significantly. Pairwise post-hoc comparisons for categorical variables were performed using two-sided Fisher exact tests with Bonferroni correction. Monte Carlo chi-square *p*-values were used for omnibus comparisons because of sparse expected cell counts. Effect size is reported as epsilon-squared for the Kruskal–Wallis test and Cramer’s *V* for categorical variables.

No statistically significant differences were observed across treatment groups in age, sex distribution, income status, or diabetes duration. Educational level and smoking status differed significantly across treatment groups (*p* = 0.003 and *p* < 0.001, respectively). Post-hoc comparisons showed that the proportion of participants with secondary education was higher in the insulin-only group than in both the oral medication and combination therapy groups. For smoking status, the combination therapy group had a lower proportion of current smokers than both the oral medication and insulin-only groups, whereas the latter two groups did not differ significantly. With regard to the duration of diabetes, no significant difference was observed between the treatment groups when comparing those with a duration of ≤6 months and those with a duration of >6 months (*p* = 0.214). The majority of participants in all treatment groups had diabetes for more than 6 months.

### 3.2 Anthropometric and dietary characteristics

Anthropometric and dietary characteristics according to treatment regimen are shown in Table 2. Body weight and BMI differed significantly across treatment groups. Post-hoc comparisons showed that the insulin-only group had significantly lower body weight and BMI than both the oral medication and combination therapy groups (*p* < 0.05 for all).

**Table 2:** Anthropometric and dietary characteristics according to diabetes treatment regimen.

Variable	Oral medication med (IQR)	Insulin only med (IQR)	Combination therapy med (IQR)	Total med (IQR)	*p*	Effect size
Weight (kg)	80.00 (70.00–89.00)^a^	67.50 (66.50–70.00)^b^	80.00 (73.00–92.00)^a^	77.80 (68.00–88.90)	<0.001	0.108
BMI (kg/m^2^)	30.32 (27.22–33.31)^a^	23.77 (22.86–26.87)^b^	30.86 (25.95–33.90)^a^	29.14 (25.45–32.81)	<0.001	0.173
Energy (kcal)	1569.85 (1170.30–1996.52)	1265.85 (1045.88–1757.65)	1284.10 (977.05–1686.30)	1467.30 (1132.70–1832.45)	0.041	0.022
Carbohydrate (g)	169.75 (143.75–221.60)^a^	135.90 (97.78–202.27)^b^	131.90 (116.20–160.20)^b^	157.80 (119.60–210.15)	<0.001	0.073
Carbohydrate (%)	48.00 (38.00–53.00)	45.50 (38.25–52.50)	42.00 (38.00–50.00)	46.00 (38.00–52.00)	0.084	0.015
Protein (g)	57.30 (45.67–80.42)	50.80 (33.10–67.42)	62.90 (41.90–72.75)	60.00 (42.85–79.20)	0.120	0.011
Protein (%)	16.00 (13.75–19.00)	16.00 (12.75–19.25)	18.00 (14.00–20.00)	17.00 (14.00–20.00)	0.255	0.004
Fat (g)	62.25 (39.50–86.00)	54.80 (48.25–68.90)	64.80 (40.95–83.25)	62.00 (42.20–84.00)	0.468	0.000
Fat (%)	36.00 (30.75–42.00)	40.00 (33.00–43.25)	39.00 (34.00–45.00)	37.00 (32.00–43.00)	0.134	0.010
Water (mL)	1200.00 (1000.00–2000.00)^a^	1550.00 (1000.00–2525.00)^b^	2000.00 (1500.00–2000.00)^b^	1500.00 (1000.00–2000.00)	<0.001	0.092

Values are presented as median (interquartile range). Different superscript letters indicate significant pairwise differences after Bonferroni correction. Effect size is reported as epsilon-squared.

Reported energy intake differed across treatment groups in the overall comparison (*p* = 0.041, *ε*^2^ = 0.022); however, no pairwise comparison remained statistically significant after adjustment. Carbohydrate intake in grams was significantly higher in the oral medication group than in both the insulin-only and combination therapy groups (*p* < 0.001). No significant differences were observed in carbohydrate percentage, protein intake, or fat intake (*p* > 0.05). Water intake differed significantly across groups, with both the insulin-only and combination therapy groups reporting higher intake than the oral medication group (*p* < 0.001).

### 3.3 Glycaemic control indicators

Glycaemic control parameters are presented in Table 3. FPG differed significantly across treatment groups in the overall Kruskal–Wallis test (*p* = 0.025, *ε*^2^ = 0.027); however, no pairwise comparison remained statistically significant after Bonferroni correction. Descriptively, median FPG was highest in the insulin-only group and lowest in the oral medication group. HbA1c levels differed significantly across treatment groups (*p* < 0.001), with the highest median HbA1c observed in the insulin-only group, followed by the combination therapy group and the oral medication group.

**Table 3:** Glycaemic control indicators according to diabetes treatment regimen.

Variable	Oral medication med (IQR)	Insulin only med (IQR)	Combination therapy med (IQR)	Total med (IQR)	*p*	Effect size
FPG (mg/dL)	181.00 (141.00–243.50)	259.00 (151.50–293.75)	219.00 (154.00–292.50)	198.00 (146.00–266.50)	0.025	0.027
HbA1c, %	8.18 (7.00–10.03)^a^	12.63 (11.25–13.11)^b^	9.74 (7.92–12.29)^c^	9.33 (7.48–12.15)	<0.001	0.193
HbA1c < 7.0%	25 (23.1)	0 (0.0)	10 (15.9)	35 (17.2)	0.009	0.215
HbA1c ≥ 7.0%	83 (76.9)	32 (100.0)	53 (84.1)	168 (82.8)		

Continuous variables are presented as median (interquartile range), and categorical variables are presented as *n* (%). Different superscript letters indicate significant pairwise differences after Bonferroni correction. Effect size is reported as epsilon-squared for Kruskal–Wallis tests and Cramer’s *V* for chi-square tests.

The proportion of participants with HbA1c < 7.0% also showed significant differences between treatment groups. Whilst no participants in the insulin-only group had an HbA1c value <7.0, 23.1% of those in the oral medication group and 15.9% of those in the combination therapy group were below this threshold (*p* = 0.009).

### 3.4 Hedonic hunger and eating-disorder symptom dimensions

Comparisons of hedonic hunger and eating-disorder symptom dimensions across treatment regimens are shown in Table 4. The PFS food available subscale differed significantly across treatment groups, with higher scores in the insulin-only group than in the oral medication group (*p* = 0.022). The PFS food present subscale also differed significantly, with higher scores in the oral medication group than in the combination therapy group (*p* = 0.021). No significant differences were observed in the PFS food tasted subscale or total PFS score (*p* = 0.828 and *p* = 0.817, respectively).

**Table 4:** Hedonic hunger and eating-disorder symptom dimensions according to diabetes treatment regimen.

Variable	Oral medication med (IQR)	Insulin only med (IQR)	Combination therapy med (IQR)	Total med (IQR)	*p*	Effect size
Hedonic hunger						
PFS: food available	3.00 (2.25–4.00)^a^	3.88 (3.00–4.00)^b^	3.50 (2.38–4.00)^ab^	3.25 (2.50–4.00)	0.022	0.028
PFS: food present	3.75 (2.00–4.25)^a^	2.50 (2.00–3.25)^ab^	2.50 (1.75–4.00)^b^	3.00 (2.00–4.00)	0.021	0.029
PFS: food tasted	4.00 (2.80–4.25)	4.00 (3.50–4.00)	4.00 (3.40–4.00)	4.00 (2.90–4.20)	0.828	0.000
PFS total score	3.32 (2.58–4.13)	3.40 (3.07–4.00)	3.31 (2.81–3.85)	3.33 (2.76–4.00)	0.817	0.000
Eating-disorder symptom dimensions						
EDE-Q-13: eating restraint	0.00 (0.00–3.67)^a^	0.00 (0.00–0.42)^b^	0.00 (0.00–2.00)^ab^	0.00 (0.00–2.33)	0.026	0.026
EDE-Q-13: shape/weight over-evaluation	0.25 (0.00–5.25)	0.00 (0.00–2.00)	0.00 (0.00–5.00)	0.00 (0.00–5.00)	0.075	0.016
EDE-Q-13: body dissatisfaction	1.00 (0.00–5.50)^a^	0.00 (0.00–2.38)^b^	0.00 (0.00–5.50)^ab^	0.00 (0.00–5.00)	0.037	0.023
EDE-Q-13: bingeing	1.83 (0.33–4.00)	1.83 (0.00–3.75)	1.00 (0.00–2.00)	1.33 (0.00–4.00)	0.078	0.016
EDE-Q-13: purging	0.00 (0.00–0.00)^a^	0.00 (0.00–0.00)^ab^	0.00 (0.00–0.00)^b^	0.00 (0.00–0.00)	0.005	0.043
EDE-Q-13: total score	1.31 (0.37–2.79)^a^	0.46 (0.00–2.00)^ab^	0.77 (0.00–2.00)^b^	1.00 (0.23–2.50)	0.009	0.038

Values are presented as median (interquartile range). Different superscript letters indicate significant pairwise differences after Bonferroni correction. Effect size is reported as epsilon-squared. PFS, Power of Food Scale; EDE-Q-13, Eating Disorder Examination Questionnaire.

Regarding eating-disorder symptom dimensions, EDE-Q-13 eating restraint, body dissatisfaction, purging, and total EDE-Q-13 score differed significantly across treatment groups (*p* = 0.026, *p* = 0.037, *p* = 0.005, *p* = 0.009, respectively). Eating restraint and body dissatisfaction scores were higher in the oral medication group than in the insulin-only group. Total EDE-Q-13 score was higher in the oral medication group than in the combination therapy group. Although purging scores differed significantly across groups, the median values were zero in all treatment groups; therefore, this finding should be interpreted cautiously. Although several between-group differences in PFS and EDE-Q-13 dimensions reached statistical significance, the corresponding effect sizes were small (*ε*^2^ = 0.023–0.043).

### 3.5 Correlations of hedonic hunger and eating-disorder symptom dimensions with glycaemic control indicators

Spearman’s correlation analyses are presented in Table 5. The observed correlations were generally weak. The PFS food available subscale was weakly and positively correlated with both HbA1c and FPG (*ρ* = 0.169, *p* = 0.016 and *ρ* = 0.207, *p* = 0.003, respectively). PFS food present, PFS food tasted, and PFS total score were also weakly and positively correlated with FPG (*ρ* = 0.172, *p* = 0.014; *ρ* = 0.210, *p* = 0.003; and *ρ* = 0.237, *p* < 0.001, respectively). Among the EDE-Q-13 measures, eating restraint and the total score were weakly and inversely correlated with HbA1c (*ρ* = −0.189, *p* = 0.007 and *ρ* = −0.157, *p* = 0.025, respectively).

**Table 5:** Correlations of hedonic hunger and eating-disorder symptom dimensions with glycaemic control indicators.

Variable	HbA1c *ρ*	HbA1c *p*	FPG *ρ*	FPG *p*
PFS: food available	0.169	0.016	0.207	0.003
PFS: food present	−0.033	0.639	0.172	0.014
PFS: food tasted	0.052	0.462	0.210	0.003
PFS total score	0.080	0.255	0.237	<0.001
EDE-Q-13: eating restraint	−0.189	0.007	−0.070	0.323
EDE-Q-13: shape/weight over-evaluation	−0.088	0.209	−0.005	0.939
EDE-Q-13: body dissatisfaction	−0.089	0.207	0.001	0.989
EDE-Q-13: bingeing	−0.069	0.330	0.069	0.328
EDE-Q-13: purging	−0.033	0.644	−0.009	0.901
EDE-Q-13 total score	−0.157	0.025	−0.009	0.899

Correlations were assessed using Spearman’s rank correlation coefficient. PFS, Power of Food Scale; EDE-Q-13, Eating Disorder Examination Questionnaire; FPG, Fasting Plasma Glucose.

### 3.6 Hierarchical linear regression analysis for HbA1c

Hierarchical linear regression analyses examining variables associated with HbA1c are presented in Table 6. Model 1, which included age, sex, BMI, diabetes duration, and treatment regimen, explained 23.6% of the variance in HbA1c (*r*^2^ = 0.236, adjusted *r*^2^ = 0.212, *p* < 0.001). The addition of smoking status and educational level in Model 2 increased the explained variance to 26.6% (Δ*r*^2^ = 0.030; robust block *p* = 0.030).

**Table 6:** Hierarchical linear regression models for HbA1c.

a. Model summary				
Model	*r* ^2^	Adjusted *r*^2^	Δ*r*^2^	Robust block *p*-value
Model 1: treatment regimen, age, sex, BMI, diabetes duration	0.236	0.212	–	–
Model 2: model 1 + smoking status and educational level	0.266	0.232	0.030	0.030
Model 3: model 2 + PFS food available	0.272	0.234	0.006	0.202
Model 4: model 3 + EDE-Q-13 total score	0.272	0.230	<0.001	0.750

b. Final model coefficients					
Variable	*b*	Robust SE	Standardized *β*	95% CI	*p*
Age, years	−0.041	0.022	−0.152	−0.085 to 0.003	0.065
Male sex	−0.491	0.445	−0.087	−1.369 to 0.387	0.272
BMI, kg/m^2^	−0.081	0.036	−0.189	−0.152 to −0.011	0.024
Diabetes duration ≤6 months	−0.759	0.768	−0.069	−2.273 to 0.756	0.325
Insulin-only regimen	2.621	0.526	0.354	1.584 to 3.658	<0.001
Combination regimen	1.157	0.430	0.198	0.308 to 2.005	0.008
Current smoker	0.378	0.454	0.059	−0.517 to 1.274	0.406
Primary education	−0.909	0.391	−0.164	−1.681 to −0.137	0.021
Secondary education or higher	−0.525	0.735	−0.059	−1.976 to 0.925	0.476
PFS food available subscale	0.223	0.171	0.086	−0.114 to 0.559	0.193
EDE-Q-13 total score	−0.042	0.133	−0.022	−0.304 to 0.219	0.750

*b*, unstandardized regression coefficient; SE, standard error; *β*, standardized regression coefficient; CI, confidence interval; BMI, body mass index; PFS, Power of Food Scale; EDE-Q-13, 13-item Eating Disorder Examination Questionnaire–Short Form. HC3 heteroscedasticity-robust standard errors and 95% confidence intervals are reported. Reference categories were oral medication only for treatment regimen, female sex, non-smoking status, illiteracy for educational level, and >6 months for diabetes duration.

The addition of the PFS food available subscale in Model 3 produced only a small increase in explained variance (Δ*r*^2^ = 0.006), and its contribution was not statistically significant (*b* = 0.223, 95% CI: −0.114 to 0.559, *p* = 0.193). Adding the EDE-Q-13 total score in Model 4 did not meaningfully improve model performance (Δ*r*^2^ < 0.001; *b* = −0.042, 95% CI: −0.304 to 0.219, *p* = 0.750). The final model explained 27.2% of the variance in HbA1c (adjusted *r*^2^ = 0.230, robust model *p* < 0.001).

In the final model, participants receiving insulin-only therapy had HbA1c values that were, on average, 2.62 percentage points higher than those receiving oral medication only (*b* = 2.621, 95% CI: 1.584–3.658, *p* < 0.001). Combination therapy was also associated with higher HbA1c compared with oral medication only (*b* = 1.157, 95% CI: 0.308–2.005, *p* = 0.008). BMI was inversely associated with HbA1c (*b* = −0.081, 95% CI: −0.152 to −0.011, *p* = 0.024). Participants with primary education had lower HbA1c than those classified as illiterate (*b* = −0.909, 95% CI: −1.681 to −0.137, *p* = 0.021). Neither the PFS food available subscale nor the EDE-Q-13 total score was independently associated with HbA1c after adjustment for treatment regimen and the other covariates.

## 4 Discussion

This study examined hedonic hunger and eating-disorder symptom dimensions in relation to glycaemic control across different treatment regimens in adults with T2DM. Several behavioural dimensions differed across treatment groups; however, the corresponding effect sizes were small. Weak bivariate associations were also observed between selected PFS and EDE-Q-13 dimensions and glycaemic indicators. Importantly, these associations did not persist in the fully adjusted hierarchical regression model: neither the PFS food available subscale nor the EDE-Q-13 total score was independently associated with HbA1c after treatment regimen and relevant demographic and clinical factors were considered. In contrast, treatment regimen remained strongly associated with HbA1c, with higher values among participants receiving insulin-only or combination therapy than among those receiving oral medication alone. BMI was inversely associated with HbA1c. Taken together, these findings suggest that eating-related behavioural characteristics may vary across treatment contexts but should be interpreted within the broader clinical heterogeneity of individuals with T2DM rather than as isolated determinants of glycaemic control.

### 4.1 Glycaemic control, treatment regimen, and BMI

The marked differences in HbA1c across treatment groups are consistent with the clinical context in which insulin therapy is commonly introduced when glycaemic control cannot be adequately maintained with oral medication alone. The persistence of higher HbA1c in both the insulin-only and combination therapy groups after adjustment supports the interpretation that treatment regimen may function as a marker of underlying disease complexity, previous glycaemic control, and treatment indication rather than representing a direct adverse effect of insulin therapy. Accordingly, the present cross-sectional findings should not be interpreted as indicating that insulin treatment itself results in poorer glycaemic control. This distinction is particularly important because treatment selection in routine clinical practice is not random and is inherently influenced by disease progression and previous treatment response.

An unexpected finding was the inverse association between BMI and HbA1c. Previous studies have frequently reported positive associations between higher BMI and poorer glycaemic control in individuals with T2DM (Deng et al., 2025; Boye et al., 2021), although the relationship is not consistent across populations (Lin, 2024). Several explanations may account for the inverse pattern observed here. First, the insulin-only group had both the lowest BMI and the poorest glycaemic profile, suggesting that the association may partly reflect differences in clinical phenotype and treatment indication. In individuals with pronounced or prolonged hyperglycaemia, glycosuria and catabolic metabolism may contribute to unintentional weight loss, making lower body weight a potential consequence rather than a cause of poorer glycaemic control. Reverse causality is therefore plausible. In addition, disease severity, treatment history, residual insulin secretion, and unmeasured changes in body weight may have contributed to this relationship. The finding should consequently be regarded as an unexpected observational association rather than evidence that higher BMI confers better glycaemic control.

### 4.2 Hedonic hunger and treatment context

The PFS food available and food present subscales differed across treatment groups, whereas the food tasted and total PFS scores did not. The higher food available score in the insulin-only group may indicate greater responsiveness to the anticipated availability of palatable foods within this treatment context. Such responsiveness may be behaviourally relevant because food availability and food-related cues can influence attention, motivation, and self-regulation even before food is physically encountered. Nevertheless, the small effect sizes observed for these differences indicate that treatment regimen accounted for only a limited proportion of variation in hedonic-hunger dimensions.

Insulin-related appetite regulation is complex. Peripheral insulin responses have been associated with appetite sensations and food intake (Flint et al., 2007), whereas central insulin signalling may also exert anorexigenic effects (Kullmann et al., 2016). In clinical practice, insulin treatment additionally introduces behavioural demands related to meal timing, carbohydrate intake, and prevention of hypoglycaemia. These factors could potentially alter food vigilance or responsiveness to food cues without necessarily representing greater hedonic hunger. Therefore, the observed group differences should not be attributed directly to the pharmacological action of insulin.

The weak positive correlations between selected PFS dimensions and glycaemic indicators are broadly consistent with previous reports linking greater hedonic hunger with less favourable glycaemic outcomes (Cheung et al., 2018; Schulte et al., 2020). However, an important finding of the revised analysis was that the PFS food available subscale was no longer independently associated with HbA1c after treatment regimen and other covariates were included. This suggests that the bivariate relationship may partly reflect differences in clinical and treatment characteristics rather than an independent behavioural effect. Furthermore, the cross-sectional design does not establish directionality. Poor glycaemic control and the intensified dietary demands accompanying more complex diabetes management may themselves influence food-related attention, dietary vigilance, or reward responsiveness. Thus, mechanisms involving greater intake of palatable foods remain plausible but were not directly tested in the present study.

### 4.3 Eating-disorder symptom dimensions and behavioural self-regulation

Differences were also observed in several EDE-Q-13 dimensions, including eating restraint, body dissatisfaction, purging, and total score, although these effects were small. Eating restraint and body dissatisfaction were higher in the oral medication group than in the insulin-only group, while total EDE-Q-13 scores were higher in the oral medication group than in the combination therapy group. These patterns should be interpreted cautiously because the EDE-Q-13 assesses dimensions of eating-disorder psychopathology rather than adaptive diabetes self-management (Esin and Ayyildiz, 2022).

In particular, the inverse correlation between eating restraint and HbA1c should not be interpreted as evidence that greater restraint is necessarily beneficial. Dietary management of T2DM requires ongoing regulation of food selection, portion size, and carbohydrate intake, and some degree of dietary control may therefore reflect adaptive diabetes-related vigilance. However, dietary restraint is not a homogeneous construct; flexible control has been associated with more favourable behavioural outcomes, whereas rigid restraint has been linked to greater food- and shape-related preoccupation and less favourable weight-control outcomes (Westenhoefer, 1991; Westenhoefer et al., 2013). Thus, the EDE-Q-13 restraint score cannot clearly distinguish adaptive dietary self-regulation from rigid or potentially maladaptive restriction in this clinical context. The present findings therefore support cautious dimensional interpretation rather than viewing greater restraint as inherently favourable for glycaemic management.

Similarly, higher body dissatisfaction among participants receiving oral medication may partly reflect their higher BMI rather than treatment regimen itself. In adults with T2DM, weight status has been reported as a strong correlate of body dissatisfaction (August et al., 2023). The statistically significant difference in purging also warrants caution because median scores were zero in all treatment groups, indicating limited absolute symptom burden despite statistical significance. These findings emphasize the importance of considering effect magnitude and score distributions alongside *p*-values.

### 4.4 Dietary intake and behavioural context

Differences in recent dietary intake were observed primarily for carbohydrate and water intake, whereas protein and fat intake did not differ significantly across treatment groups. Because dietary assessment was based on a single 24-h recall, these findings should be interpreted as estimates of recent intake rather than habitual dietary behaviour; a single recall is recognized as a poor estimator of an individual’s long-term usual intake because of substantial within-person day-to-day variation (Dodd et al., 2006). The higher carbohydrate intake reported in the oral medication group may reflect differences in total food intake, dietary counselling, or treatment-related self-management, but the present design does not permit these possibilities to be distinguished.

More broadly, eating behaviour in T2DM may reflect interactions among physiological appetite, food-cue responsiveness, diabetes-related dietary vigilance, treatment demands, and self-regulatory processes (Cheung et al., 2018; Schulte et al., 2020; Gal et al., 2024; Freitag et al., 2025). Psychological factors may also contribute. Diabetes-related distress has been associated with poorer glycaemic control and reduced self-care behaviours, while fear of hypoglycaemia can influence food-related decisions and diabetes self-management (Przezak et al., 2022; Ajele and Idemudia, 2025). These dimensions were not directly measured in the present study but may partly explain why behavioural scores differed across treatment contexts without showing strong independent associations with HbA1c.

### 4.5 Multivariable findings and clinical implications

The hierarchical analysis further emphasizes the multifactorial nature of glycaemic control. The final model explained 27.2% of the variance in HbA1c, while the adjusted *r*^2^ was 0.230, indicating that a substantial proportion of interindividual variability remained unexplained. Glycaemic control is influenced by factors beyond those assessed in the present study. For example, medication adherence has been associated with better glycaemic control in adults with T2DM (Piragine et al., 2023), and evidence from randomized trials demonstrates that structured physical activity can meaningfully reduce HbA1c (Gallardo-Gómez et al., 2024). Psychosocial factors, including diabetes-related distress, are also associated with glycaemic control and self-care behaviours (Ajele and Idemudia, 2025). Consequently, the behavioural measures examined here should be considered components of a broader biopsychosocial framework rather than dominant determinants of HbA1c.

The findings nevertheless support consideration of eating-related behavioural characteristics during nutritional assessment, particularly when individuals report difficulty regulating intake in response to food cues, excessive dietary restriction, body-related concerns, or recurrent challenges with diabetes self-management. Such assessment should not imply that elevated PFS or EDE-Q-13 scores directly cause poorer glycaemic control. Rather, identifying relevant behavioural concerns may help guide individualized nutritional and behavioural support. Evidence from systematic reviews suggests that motivational interviewing can improve metabolic and psychosocial outcomes in diabetes (Bilgin et al., 2022), while cognitive-behavioural interventions may improve glycaemic and psychological outcomes in adults with diabetes (Uchendu and Blake, 2017). Prospective studies incorporating repeated behavioural assessments, detailed treatment information, diabetes distress, hypoglycaemia experiences, habitual dietary intake, and longitudinal HbA1c measurements are needed to clarify the temporal and mechanistic relationships among treatment context, eating behaviour, and glycaemic control.

### 4.6 Strengths and limitations of the study

This study has several strengths, including the simultaneous assessment of hedonic hunger, eating-disorder symptom dimensions, dietary intake, and objective glycaemic indicators across three commonly used treatment regimens. The use of validated Turkish versions of the PFS and EDE-Q-13 and a hierarchical regression model incorporating relevant clinical and demographic factors further strengthened the analysis.

Several limitations should be considered. The cross-sectional design precludes causal inference, and treatment allocation was not randomized, leaving the possibility of confounding by indication. Specific drug classes, insulin types, medication doses, and treatment adherence were not recorded, while diabetes duration was assessed categorically. Dietary intake was based on a single 24-h recall, and behavioural measures were self-reported. The single-centre outpatient setting may also limit generalizability. In addition, a formal screening log was not maintained, preventing calculation of the participation rate and full assessment of selection bias. The highly unbalanced distribution of diabetes duration, with relatively few participants in the ≤6-month category, limited its ability to adequately represent diabetes chronicity and may have reduced the precision of its estimate in the multivariable model. Finally, a substantial proportion of the variability in HbA1c remained unexplained, suggesting contributions from unmeasured clinical, behavioural, and psychosocial factors.

## 5 Conclusion

This study showed that hedonic hunger and eating-disorder symptom dimensions varied modestly across treatment regimens in adults with T2DM, while glycaemic control differed more substantially between treatment groups. Although selected PFS and EDE-Q-13 dimensions showed weak bivariate associations with FPG and HbA1c, neither the PFS food available subscale nor the EDE-Q-13 total score remained independently associated with HbA1c after adjustment for treatment regimen and relevant demographic and clinical factors. In contrast, insulin-only and combination therapy were associated with higher HbA1c compared with oral medication alone, likely reflecting differences in disease complexity and treatment indication rather than direct treatment effects. The inverse association between BMI and HbA1c should likewise be interpreted cautiously in view of the cross-sectional design and potential reverse causality. Overall, these findings suggest that eating-related behavioural characteristics may contribute to the broader clinical context of T2DM management but are unlikely to act as major independent determinants of glycaemic control. Prospective studies incorporating detailed treatment, behavioural, and psychosocial measures are needed to clarify these relationships.

## Data Availability

The raw data supporting the conclusions of this article will be made available by the authors, without undue reservation.
